# Effects of non-thermal food processing techniques on the composition, bioaccessibility and shelf-life of bioactive compounds in a fruit juice blend

**DOI:** 10.1016/j.fochx.2025.103000

**Published:** 2025-09-08

**Authors:** Gulay Ozkan, Manolya Eser Oner, Annik Fischer, Andreas Juadjur, Kemal Aganovic, Gerald Dräger, Esra Capanoglu, Tuba Esatbeyoglu

**Affiliations:** a Department of Food Engineering, Faculty of Chemical and Metallurgical Engineering, İstanbul Technical University, 34469 Maslak, Istanbul, Türkiye; b Department of Food Engineering, Faculty of Engineering, Alanya Alaaddin Keykubat University, 07425 Alanya, Antalya, Türkiye; c Department of Molecular Food Chemistry and Food Development, Institute of Food and One Health, Gottfried Wilhelm Leibniz University of Hannover, Am Kleinen Felde 30, 30167 Hannover, Germany; d DIL German Institute of Food Technologies e.V., Prof.-von-Klitzing-Str. 7, 49610 Quakenbrück, Germany; e Institute of Food Quality and Food Safety, University of Veterinary Medicine Hannover, Foundation, Bischofsholer Damm 15, 30173, Hannover, Germany; f Institute of Organic Chemistry, Gottfried Wilhelm Leibniz University Hannover, Schneiderberg 1b, 30167 Hannover, Germany

**Keywords:** High pressure processing, Pulsed electric field, Phenolic compounds, Anthocyanins, Antioxidant activity, Shelf-life, Digestion

## Abstract

In this study, the effects at various conditions of high-pressure processing (HPP) at 600 MPa/3 min, 500 MPa/5 min, 500 MPa/10 min, pulsed electric field (PEF) at 120 kJ/L-24 kV/cm, 100 kJ/L-20 kV/cm, 100 kJ/L-15 kV/cm and thermal treatment (TT) at 80 °C/ 30 min on the stability, digestibility properties, and durability during shelf-life of phenolic compounds in fruit juice blend (21 % kiwi, 10.5 % mango, 37 % orange, 31.5 % blueberry) have been investigated. Results of this study indicated that the highest content on bioactive substance and antioxidant capacity values were obtained for HPP treatment at 600 MPa/3 min and for PEF treatment at 120 kJ/L-24 kV/cm. After *in vitro* digestion, the highest total phenolic content (TPC), total flavonoid content (TFC) and total anthocyanin content (TAC) values were detected in the PEF treated samples. Moreover, the bioactive content of the non-thermal-treated samples was found to be protected as that of thermal-treated sample during storage, except for individual anthocyanin and vitamin C contents in HPP-treated samples.

## Highlights

• The highest bioactive substance and antioxidant capacity were obtained at 600 MPa/3 min for HPP treatment.
• The highest bioactive substance and antioxidant capacity were obtained at 120 kJ/L-24 kV/cm for PEF treatment.
• The highest phenolic and antioxidant bioaccessibility was found with PEF treatment.
• Hesperidin is the most abundant phenolic compound identified in the fruit juice blends.
• HPP and PEF treatments are promising technologies to enhance the nutritional quality and shelf-life of fruit juice blends.

## 1 Introduction

Consumers' changing lifestyles and eating habits have led to production of affordable, healthy, and quick sources of nutrition such as packed fruit juices. Globally, there has been an increased demand on sugar-free and preservative-free natural fruit juices. As they are accepted as functional food products, antioxidant and antimicrobial properties of phenolic compounds in fruits have positive effects on human health such as reducing risk of cancer, cardiovascular and neurogenerative diseases (Durazzo et al., 2019). Yet, phenolic compounds found in complex food matrix, efficacy in the human body may vary based on digestibility (Ozkan et al., 2020). For this reason, researches revealed that the amount of bioavailable bioactive compound is more important than the amount present in the food product. Bioaccessibility is an important factor for the bioactivity of polyphenols in gastrointestinal digestion; bioaccessibility is defined as the available biological fraction from food matrix for absorption in gastrointestinal digestion (Jafari & McClements, 2017). With simple, inexpensive and convenient applications, *in vitro* methods have been used to estimate bioaccessibility of compounds at different digestion phases as an alternative to *in vivo* methods (Alminger et al., 2014).

Conventional pasteurization method has been commonly used in fruit juice industry, which can deteriorate nutritional quality and fresh-like characteristics of product. Alternatively, nonthermal technologies often provide nutritionally superior options to thermal pasteurization with safe products by largely retaining their nutritional and sensorial properties (Bevilacqua et al., 2018). High pressure processing (HPP) has proved to be an effective nonthermal technology, which uses water as a pressure transmission medium with a pressure range varies between 100 and 800 MPa. It is used in the food industry as a preservation technique which prolongs the shelf-life of several products by ensuring high quality levels in terms of vitamins and nutrition (Aganovic et al., 2021). There are several factors that affect the bioaccessibility of bioactive compounds including type of processing technique, compound type, food matrix, and other components (Cilla et al., 2018). Some researchers observed increase in bioaccessibility of bioactive compounds in HPP treated food products, which might be due to nutrient release caused by cellular disruption. Treatment of 600 MPa HPP for 6 min resulted in higher phenolics bioaccessibility (35.12 %) was in the cloudy hawthorn berry juice compared to control (30.53 %) and thermal treated at 65 °C for 30 min (31.14 %) samples (Lou et al., 2022). In other research study, there was no significant difference determined in individual anthocyanin content of cloudy pomegranate juice after intestinal digestion when treated with high pressure or thermal (Yuan et al., 2022). In the same study, when compared to untreated samples, HPP did not affect the antioxidant capacity of cloudy pomegranate juice after intestinal digestion with 2,2′-azinobis-(−3 ethylbenzothiazoline-6-sulfononic acid) diammonium salt (ABTS) and 2,2-diphenyl-1-picrylhydrazyl (DPPH) values lower than 0.40 mmol Trolox equivalents (TE)/L and 0.30 mmol TE/L, respectively.

Pulsed electric field (PEF) has been used commercially as a processing technique by pumping food product between two electrodes, where high intensity electric field is applied in the form of very short pulses. Depending on the application, the electric field strength changes between 0.1 kV/cm to typically 3 kV/cm for cell disintegration and modifying food structure, and 10–30 kV/cm for electroporation of microbial cells. Pulse duration is typically from μsec to msec (Brito & Silva, 2024). Some studies showed that PEF technology did not affect the content of phenolic compounds compared to control samples, as in the case of applying PEF under 19 kV/cm field strength, 1.25 kHz frequency, 2 μs pulse width, 181 μs treatment time to orange juice (Jin & Aboelhaggag, 2022) and 20 kV/cm field strength, 0.066 Hz frequency, 0.17 μs pulse width to chokeberry juice (Oziembłowski et al., 2022). However, other researchers found notable improvement in phenolic compounds of raspberry juice by 35 to 74 % depending on the intensity and frequency of the electric field (Buitimea-Cantúa et al., 2022). Similar results were obtained in spinach juice and wheatgrass juice with 8.49 % and 5.35 % increases in phenolics compared to control, respectively (Ahmed et al., 2021; Faisal Manzoor et al., 2021). Further studies by Rodríguez-Roque et al. (2015a) proved that PEF treatment (35 kV/cm, 1800 μs) improved bioaccessibility of vitamin C, phenolic compounds and antioxidant capacity compared to thermal processing (90 °C, 60 s) in fruit-based beverages. In other study, significant enhancement was observed in bioaccessibility of total phenolic compounds (37 %) and total anthocyanins (16 %) of mixed fruit juice (50.75 % papaya, 19.25 % mango, 30 % *Stevia rebaudina* infusion) after PEF treatment (25 kV/cm and 50–400 pulses) compared to untreated samples, however; there was no vitamin C found in juice samples after intestinal digestion (Buniowska et al., 2017).

The aim of the present study was to determine the effect of high-pressure processing (HPP), pulsed electric field (PEF), and thermal pasteurization (TT) on the content of bioactive compounds (total phenolic content, TPC; total flavonoid content, TFC; total anthocyanin content, TAC; antioxidant capacity, AOX; vitamin C as well as individual phenolic acids, flavonoids and anthocyanins) in fruit juice blends (21 % kiwi, 10.5 % mango, 37 % orange, 31.5 % blueberry) before and after processing. Secondly, the changes in the bioactive content of the fruit juice blends were assessed during *in vitro* digestion. Thirdly, effects of processing on the shelf-life of fruit juice blends stored at 4 °C for 6 months were investigated. Samples were evaluated in terms of TPC, TFC, TAC, AOX, vitamin C and individual anthocyanin levels. The phenolic profile of fruit juice blend sample was unveiled by using UPLC-QTOF-MS/MS.

## 2 Materials and methods

### 2.1 Materials

Orange, kiwi, blueberry and mango fruits purchased from a local supermarket (Quakenbrück, Germany). All chemicals and reactive, analytical or HPLC level were obtained from Sigma-Alderich (Steinheim, Germany), it was not mentioned unless it is obtained from different company.

### 2.2 Sample preparation

Approximately 100 L of fruit juice blend obtained from fresh kiwi (21 %), mango (10.5 %), orange (37 %), blueberry (31.5 %) by using pilot scale Fresh'n Squeeze Multi-Fruit Juicer (Elea Technology GmbH, Quakenbrück, Germany) at the Deutsches Institut für Lebensmitteltechnik e.V. (DIL e.V., Quakenbrück, Germany). This fruit juice blend was then immediately processed by thermal or non-thermal applications.

### 2.3 Processing techniques

Fruit juice blends were processed by using industrial-scale HPP unit (Wave 6000/55, Hiperbaric S.A., Burgos Spain). Here, water was used as pressure transmitting medium. Samples were subjected to three different conditions as 500 MPa for 5 min (HPP1), 500 MPa for 10 min (HPP2) and 600 MPa for 3 min (HPP3) at ambient temperature (20 ± 2 °C) (Yildiz et al., 2023). PEF treatment was conducted on a pilot scale system (HVP 5 kW, Elea GmbH, Quankenbrück, Germany). The system was connected to a spiral pump (Seepex GmbH, Bottrop, Germany) with a flowrate of 35 L/h. The PEF operated for three different conditions, specific energy inputs and electric field strengths were 100 kJ/L and 15 kV/cm (PEF1), 100 kJ/L and 20 kV/cm (PEF2) and 120 kJ/L and 24 kV/cm (PEF3). These conditions were determined based on the sample electric conductivity, PEF equipment capacity and industrial recommendations. Pulse width was 20 μs and bipolar rectangular pulses (peak-peak voltages) were applied in treatment chamber with a 10 mm electrode gap. The traditional thermal pasteurization (TT) was conducted to fruit juice blend at 80 °C for 30 min and cooled to room by using pilot scale unit **(**PT 60 Pilot Scale Waterbath, Quakenbrück, Germany). Besides, untreated fresh blend sample was described as control.

Sterile 100 mL volume PET plastic round beverage bottles (Nipak BV, Lopik, Netherlands) were used to fill the samples after PEF treatment and before HPP and TT on a clean bench. Treated samples were stored at −80 °C until further analysis. With the selected conditions recommended by the industry, HPP treated samples at 600 MPa for 3 min (HPP3), PEF treated samples at 120 kJ/L and 24 kV/cm (PEF3) and TT samples were stored under refrigeration condition (4 ± 1 °C) in order to evaluate the changes in bioactive compounds for the six months shelf-life study.

### 2.4 Physicochemical analysis of the samples

The pH and conductivity of the fruit juice blends were evaluated using a digital pH meter equipped with conductometer at 20 °C. Minolta CM-5 spectrophotometer (Konica Minolta, Langenhagen, Germany) was used to define color of fruit juice blends, in which L* ranges from lightness (100) to darkness (0), chromatic components of a* (redness to greenness) and b* (yellowness to blueness) range from +60 to −60, C* is chroma ranging from 0 (least saturation) to 60 (full saturation), and h° is hue angle ranging from 0° to 360° where 0° or 360° is red, 90° is yellow, 180° is green and 270° is blue. The chromameter was calibrated against the white plate prior to usage. During the measurements, 10 mL of the sample was placed into 60 mm disposable petri plate and L*, a*, b*, C* and h° values were recorded three times by randomly measuring bottom side of the plate at room temperature (∼20 °C).

### 2.5 Spectrophotometric assays

All spectrophotometric assays were performed by using Infinite M200 UV–visible spectrophotometer (Tecan, Crailsheim, Germany) in three replicates.

Total phenolic content (TPC) was determined using Folin-Ciocalteu reagent at 765 nm as described previously by (Singleton & Rossi, 1965) The results were expressed as mg gallic acid equivalents (GAE) per 100 mL of fruit juice or blend samples.

Total flavonoid content (TFC) assay was performed according to the method of (Dewanto et al., 2002). The measurements were applied at 510 nm. The results were expressed as mg rutin equivalents (RE) per 100 mL of fruit juice or blend samples.

The total monomeric anthocyanin content (TAC) was determined by pH differential method (Giusti & Wrolstad, 2001). Absorbances of samples diluted with pH 1.0 and pH 4.5 buffers were measured at 520 and 700 nm. The results were expressed as mg cyanidin-3-*O*-glucoside equivalents (C3G) per 100 mL of fruit juice or blend samples by using the following formula:

$$\;\text{Total monomeric anthocyanin}\;\left(\mathrm{mg}/L\right)=\left(A\;x\;\mathrm{MW}\;x\;\mathrm{DF}\;x\;1000\right)/\left(\varepsilon \;x\;l\right)$$

where A = (A_520nm_ − A_700nm_)_pH_ _1.0_ − (A_520nm_ − A_700nm_)_pH_ _4.5_, MW is the molecular weight of cyanidin-3-*O*-glucoside (449.2 g/mol), DF is the dilution factor, 1000 is the conversion factor from g to mg, ε is the molar extinction coefficient of cyanidin-3-glucoside (26,900 L/(mol.cm)), and l is the path length (cm).

The antioxidant capacities (AOX) of fruit juice blends were estimated by using the cupric ion reducing antioxidant capacity (CUPRAC) (Apak et al., 2004) and 2,2-diphenyl-1-picrylhydrazyl (DPPH) (Molyneux, 2004) assays at 450 and 517 nm, respectively. In the assays, 6-hydroxy-2,5,7,8-tetramethylchroman-2-carboxylic acid (Trolox) was used as a standard and results were expressed as mg Trolox equivalents (TE) per 100 mL of fruit juice or blend samples.

### 2.6 Identification of polyphenols by UPLC-QTOF-MS/MS

Polyphenols were identified using “liquid chromatography quadrupole time-of-flight tandem mass spectroscopy (LC-QTOF-MS) (Ozkan, Kostka, et al., 2022). Before LC-MS/MS analysis, fresh fruit juice blend was passed through 0.20 μm PTFE membrane filters. LC-MS/MS analysis was performed on a Waters Acquity UPLC system (Waters Co., Milford, MA, USA) connected to a Waters Q-Tof Premier mass spectrometer equipped with an electrospray ionisation (ESI) source. The UPLC system was equipped with a binary pump with degasser, autosampler, photodiode array detector and a column oven. For chromatographic separation, an Acquity UPLC BEH Phenyl (100*2.1 mm, 1.7 μm) column was used for the separation. Mobile phase consisting of formic acid/MQ water (1/1000, *v*/v; eluent A) and formic acid/acetonitrile (1/1000, *v/v*; eluent B) was used. The linear gradient was as follows: 0 min, 5 % B; 0–6.48 min, 35 % B; 6.48–6.77 min, 100 % B; 6.77–8.00 min, 100 % B; 8.00–8.10 min, 5 % B. The injection volume was 10 μL. aThe flow rate was set at 0.6 mL/min. The column temperature was kept at 45 °C, while the temperature of the autosampler was held at 10 °C. ESI-MS analysis was performed in both positive and negative modes. Collision energies of 15 V (for low energy) and 30 V (for high energy) was used for full-scan LC-MS in the *m/z* 100–1500. Acquisition and integration of chromatograms was performed using the Masslynx software from Waters.

### 2.7 Quantification of polyphenols using UPLC-PDA

The method for determination of phenolics by UPLC was adapted from Ozkan et al. (2021) with minor modifications. Fruit juice blend samples obtained from different processing conditions was passed through 0.20 μm PTFE membrane filters and injected into a UPLC system (Agilent 1290 Infinity II, Germany) coupled with a photodiode array detector (PDA). A Luna C18 (250*4.6 mm, 5 μm) column from Phenomenex (Aschaffenburg, Germany) was used as stationary phase. The following solvents with a flow rate of 1 mL/min and injection volume of 10 μL was used for spectral measurements: Trifluoroacetic acid (TFA)/distilled water (1:1000, *v*/v, eluent A) and TFA/acetonitrile (1:1000, v/v; eluent B). The linear gradient will be as follows: The linear gradient was as follows: 0 min, 5 % B; 0–6.48 min, 35 % B; 6.48–6.77 min, 100 % B; 6.77–8.00 min, 100 % B; 8.00–8.10 min, 5 % B. Phenolic compounds were quantified by using their authentic standards. For calibration curves, chromatographic peak area of the standards *versus* nominal concentrations were plotted. All results were expressed as mg per 100 mL of fruit juice blend samples.

### 2.8 Anthocyanin profile evaluation

The method for quantitative HPLC-PDA determination of anthocyanin profiles was adapted from Juadjur et al. (2015) with minor modifications. Fruit juice blend samples obtained from different processing conditions were passed through 0.45 PTFE μm membrane filters and injected into a HPLC Nexera-i LC-2040C 3D Plus (Shimadzu Corporation, Kyoto, Japan) coupled with a photodiode array detector (PDA). A Phenomenex Luna C18 (250*4.6 mm, 5 μm) column was used as stationary phase. Identification of single anthocyanins was previously performed and described in (Juadjur & Winterhalter, 2012). Solvent A was water/acetonitrile/formic acid (87:3:10, *v*/*v*/v) and solvent B water/acetonitrile/formic acid (40:50:10, v/ v/v). A flow rate of 0.5 mL/min was used, together with the following gradient: 2–14 % B (0–20 min), 14 % B (20–25 min), 14–18 % B (25–40 min), 18 % B (40–45 min), 18–90 % B (45–70 min), 90–2 % B (70–80 min) and 2 % B (80–90 min). The detection wavelength for the anthocyanins was 520 nm. All results were expressed as mg per 100 mL of fruit juice blend samples.

### 2.9 Ascorbic acid/dehydroascorbic acid determination

Dehydroascorbic acid and ascorbic acid were determined using the method of Kneifel and Sommer (1985). The fruit juice blend samples were homogenized with a solution of m-phosphoric acid and acetic acid, then mixed with bidistilled water. Activated charcoal was added to the sample to convert l-ascorbic acid to dehydro-l-ascorbic acid. The mixture was then thoroughly mixed, filtered, and diluted with water and a sodium acetate solution. This solution was further mixed with a phenylenediamine solution and incubated in the dark for 16 h. After membrane filtration, the samples were injected and analyzed by high-performance liquid chromatography (HPLC Alliance Separations Module 2695, Waters Eschborn, Germany) using a LiChrospher 100 C18 column, equiped with a fluorescence detector (2475 Multi Lambda, Waters). The emission wavelength was set at 430 nm and the excitation wavelength at 350 nm. The mobile phase consisted of 50 % methanol with 92 mM sodium acetate, 4.6 mM phosphoric acid and 21 mM acetic acid under isocratic conditions at a flow rate of 0.8 mL/min. All results were expressed as mg per 100 mL of fruit juice blend samples.

### 2.10 *In vitro* gastrointestinal digestion simulation

The *in vitro* digestion procedure was performed according to the method described by Sessa et al. (2011). The only modification was the absence of the addition of saliva. The method consists of two sequential steps; an initial pepsin/HCl digestion to simulate gastric conditions, followed by a digestion with bile salts/pancreatin to simulate intestinal digestion. Briefly, 5 mL of fruit juice blend was mixed with 5 mL of phosphate buffer saline (PBS) and preincubated in a water bath at 37 °C for 15 min. Before starting gastric digestion, the pH was adjusted to 2 using 1 M HCl and porcine pepsin was then added to a final concentration of 1.3 mg/mL. The samples were incubated at 37 °C in a shaking water bath (GFL 1092, Burgwedel, Germany) at 100 rpm for 2 h. For the intestinal digestion, the pH of the digest raised to pH 5.8 with 1 M NaHCO_3_ dropwise, and 2.5 mL of pancreatin and bile salts mixture were added to final concentrations of 0.175 and 1.1 mg/mL, respectively. The pH was then adjusted to pH 6.5 with 1 M NaHCO_3_, and samples were incubated at 37 °C in a shaking water bath at 100 rpm for 2 h. After gastrointestinal digestion, the samples were cooled by immersing in an ice bath and then centrifuged at 10,000 rpm for 30 min at 4 °C (Megafuge 8R; Thermo Scientific, Darmstadt, Germany) to separate the soluble or bioaccessible fraction and the residual fraction.

A blank (without the added sample) was incubated under the same conditions to discard interferences due to the digestive enzymes and buffers used in the digestion process. All experiments were performed in triplicates.

Bioaccessible fractions of the digests were stored at −20 °C and used for spectrophotometric measurements as indicated in section 2.5 (total phenolic, flavonoid and anthocyanin contents, and antioxidant capacity) and chromatographic measurements as indicated in section 2.7.

Bioaccessbility was calculated by using the following formula:

$$\text{Bioaccessibility}\;\left(\%\right)={\left(\text{bioactive content of}\;\mathrm{ID}/\text{bioactive content of}\;\mathrm{UD}\right)}^{*}\;100$$

where bioactive content of ID means the amount of total phenolic/flavonoid/anthocyanin/antioxidant capacity in intestinal digested (ID) sample and bioactive content of UD means the amount of total phenolic/flavonoid/anthocyanin/antioxidant capacity in undigested sample (UD).

### 2.11 Shelf-life study

Fruit juice blends treated with HPP3 (600 MPa-3 min), PEF3 (120 kJ/L-24 kV/cm), and TT (80 °C-30 min) were stored at 4 ± 1 °C for 6 months and changes in bioactive compounds were evaluated monthly, as indicated in section 2.5, 2.8 and 2.9.

### 2.12 Statistical analysis

All experiments were conducted in three replication and three repeats. Results were reported as mean ± standard deviation. Treatments were compared using one-way analysis of variance (ANOVA) followed by a Tukey *post hoc* test (*p* < 0.05) by using SPSS software (version 27.0, IBM Corp., Armonk, NY, USA).

## 3 Results and discussion

### 3.1 Effect of processing on the physicochemical properties of fruit juice blend

The pH, conductivity and color changes in fruit juice blends after TT, HPP and PEF treatments were represented in Table 1. Different processing techniques did not affect the pH value of the samples, which ranged between 3.34 and 3.37. Similar result was obtained in pH of strawberry juice; no significant difference was determined after TT, HPP and PEF application (Yildiz et al., 2021). Electrical conductivity is a critical parameter in PEF processing which increased from 4168.5 μS/cm to 4181.0 μS/cm after PEF treatment; however, increase in conductivity was not significant (*p* *>* 0.05). Color values in terms of L* and C* indicated that PEF processing increased lightness and saturation in fruit juice blends compared to HPP treated and control samples (*p* *<* 0.05). In addition, PEF processing enhanced redness and yellowness in samples (*p* *<* 0.05). Other researchers reported similar effect on beetroot (Nowacka et al., 2019) and pomegranate beverage (Rios-Corripio et al., 2022) with an increase in redness (a*) and lightness (L*) after PEF treatment, respectively. This might be due to the pores generated by PEF, which enhances the diffusion of substances from intracellular to extracellular environment; thereby, anthocyanins and flavonoids become available and alters the color (Manzoor et al., 2020). On the other hand, HPP did not affect the color values of fruit juice blends compared to fresh samples. Findings from the current study were consistent with the HPP applications on avocado dressing (Oner, 2020), carrot juice (Pokhrel et al., 2019), and strawberry puree (Aaby et al., 2018). Results indicated that color quality of fruit juice blends were enhanced by PEF treatment while remained constant with HPP relative to fresh ones.

**Table 1:** Changes in pH, conductivity, and color values.

Samples^1^	pH	μS/cm	L*	a*	b*	C*	h°
**Control**	3.34 ± 0.02^a^	4168 ± 9^a^	9.62 ± 0.30^cd^	15.73 ± 0.59^c^	5.83 ± 0.23^d^	16.78 ± 0.62^cd^	20.34 ± 0.32^ab^
**TT**	3.37 ± 0.00^a^	n.d.	10.46 ± 0.31^a^	19.54 ± 0.50^a^	6.24 ± 0.25^c^	20.51 ± 0.53^ab^	17.70 ± 0.56^d^
**HPP1**	3.37 ± 0.01^a^	n.d.	9.10 ± 0.31^e^	15.97 ± 0.78^c^	5.99 ± 0.21^cd^	17.06 ± 0.78^c^	20.58 ± 0.77^a^
**HPP2**	3.35 ± 0.01^a^	n.d.	8.37 ± 0.45^f^	15.82 ± 0.25^c^	5.69 ± 0.09^d^	16.81 ± 0.25^cd^	19.79 ± 0.30^ab^
**HPP3**	3.35 ± 0.01^a^	n.d.	9.27 ± 0.18^de^	15.39 ± 0.23^c^	5.70 ± 0.08^d^	16.41 ± 0.22^d^	20.31 ± 0.35^ab^
**PEF1**	3.36 ± 0.03^a^	4181 ± 24^a^	9.88 ± 0.66^bc^	19.15 ± 0.23^ab^	6.73 ± 0.64^a^	20.31 ± 0.29^ab^	19.33 ± 1.72^bc^
**PEF2**	3.37 ± 0.01^a^	4179 ± 7^a^	10.17 ± 0.14^ab^	19.57 ± 0.48^a^	6.58 ± 0.23^ab^	20.65 ± 0.51^a^	18.57 ± 0.40^cd^
**PEF3**	3.37 ± 0.00^a^	4170 ± 20^a^	10.54 ± 0.32^a^	18.89 ± 0.26^b^	6.30 ± 0.21^bc^	19.92 ± 0.21^b^	18.46 ± 0.74^cd^

1 Data in the same column followed by the different letters are significantly (*p* *<* 0.05) different. n.d.: not determined.

### 3.2 Effect of processing on the retention of bioactive compounds in fruit juice blends

The changes in TPC, TFC, TAC and AOX of fruit juice blends after thermal (TT) and non-thermal (HPP and PEF) processing were indicated in Table 2. With the highest TPC (138.36 ± 11.07 mg GAE/100 mL) and TFC (72.54 ± 1.12 mg RE/100 mL) (*p* *<* 0.05), blueberries were the main source of phenolics among the other fruits (mango, orange, kiwi) in fruit juice blend. After HPP and PEF treatments, TPC of fruit juice blend varied from 94.42 to 96.94 mg GAE/100 mL and 93.87–104.10 mg GAE/100 mL, respectively. Even though, there was a slight increase in TPC values after non-thermal treatments, no significant difference was observed when compared to fresh (control) and thermal-treated fruit juice blends (*p* *>* 0.05). In consistent to our results, other researchers revealed that PEF did not cause considerable losses of phenolic compounds in orange juice (Jin & Aboelhaggag, 2022) and chokeberry juice (Oziembłowski et al., 2022). On the other hand, higher TFC values were obtained in both HPP and PEF treated samples than fresh (control) fruit juice blend (*p* *<* 0.05). Thermal treatment showed similar effect as HPP on flavonoids, which was significantly higher than fresh fruit juice blend (*p* *<* 0.05). Thermal treatment may affect the food matrix and microstructure with the influence of phytochemical abundance and degradation of compounds. However, it is important to highlight that TT can increase the accessibility of compounds and extraction of bioactive compounds by damaging cell walls (Barba et al., 2017). Nevertheless, PEF treatment with 120 kJ/L-24 kV/cm and 100 kJ/L-20 kV/cm had the positive impact on flavonoids of fruit juice blend. Because biological activities in flavonoids are structure dependent and they are different according to their degree of hydroxylation, polymerization and other substitutions and conjugations (Kumar & Pandey, 2013).

**Table 2:** Changes in total phenolic (TPC), total flavonoid (TFC), total monomeric anthocyanin (TAC) and vitamin C contents as well as antioxidant capacity (AOX) of fruit juice or blend samples.

Sample^1^	TPC (mg GAE/100 mL)	TFC (mg RE/100 mL)	TAC (mg C3G/L)	Vitamin C (mg/100 mL)	AOX	
					CUPRAC (mg TE/100 mL)	DPPH (mg TE/100 mL)
**Mango**	54.54 ± 4.29^d^	5.46 ± 0.30^i^	n.d.	48.00 ± 0.42^a^	111.28 ± 12.73^d^	75.29 ± 8.37^fg^
**Blueberry**	138.36 ± 11.07^a^	72.54 ± 1.12^a^	166.47 ± 6.81^a^	1.15 ± 0.02^f^	313.02 ± 60.34^a^	122.12 ± 9.00^a^
**Orange**	68.59 ± 4.73^cd^	10.36 ± 0.12^h^	n.d.	40.00 ± 0.64^b^	117.44 ± 20.48^cd^	65.71 ± 7.19^g^
**Kiwi**	71.62 ± 8.77^c^	18.48 ± 0.32^g^	n.d.	47.90 ± 0.28^a^	123.67 ± 4.98^cd^	63.88 ± 5.73^g^
**Control**	92.36 ± 7.07^b^	24.59 ± 0.30^f^	58.22 ± 4.41^d^	22.45 ± 1.59^cd^	189.85 ± 32.11^bc^	99.74 ± 11.93^bc^
**TT**	95.13 ± 8.21^b^	26.96 ± 1.06^de^	70.16 ± 2.15^bc^	23.75 ± 0.70^c^	199.55 ± 30.20^b^	109.79 ± 6.92^ab^
**HPP1**	96.41 ± 9.14^b^	25.99 ± 0.50^e^	54.66 ± 2.09^d^	21.83 ± 1.04^cde^	181.25 ± 29.49^bcd^	82.93 ± 9.34^ef^
**HPP2**	96.94 ± 5.75^b^	27.01 ± 1.40^cd^	57.87 ± 1.71^d^	18.03 ± 0.57^e^	175.63 ± 27.60^bcd^	84.05 ± 5.05^def^
**HPP3**	94.42 ± 10.52^b^	26.90 ± 0.50^de^	65.92 ± 2.26^c^	21.30 ± 0.07^cde^	186.66 ± 16.61^bc^	88.91 ± 10.52^cdef^
**PEF1**	93.87 ± 11.65^b^	27.98 ± 2.47^c^	69.83 ± 5.11^bc^	19.43 ± 0.88^de^	203.68 ± 59.41^b^	96.10 ± 8.96^bcde^
**PEF2**	104.10 ± 9.15^b^	29.80 ± 0.67^b^	72.78 ± 3.17^b^	18.78 ± 0.33^de^	228.67 ± 48.87^b^	95.37 ± 5.87^bcde^
**PEF3**	98.99 ± 10.78^b^	30.02 ± 0.91^b^	74.20 ± 8.12^b^	18.38 ± 0.45^e^	205.02 ± 29.23^b^	98.75 ± 7.05^bcd^

TPC: Total Phenolic Content, TFC: Total Flavonoid Content, TAC: Total Anthocyanin Content, AOX: Antioxidant Capacity. GAE: Gallic acid equivalents, RE: Rutin equivalents, C3G: Cyanidin-3-*O*-glucoside equivalents, CUPRAC: Cupric ion reducing antioxidant capacity, TE: Trolox equivalents, DPPH: 2,2-diphenyl-1-picrylhydrazyl, n.d.: not detected.

1 Data in the same column followed by the different letters are significantly (*p* < 0.05) different.

With regarding to the value of TAC, TT and PEF treatments significantly increased anthocyanins in fruit juice blends; however, no significant difference was observed in fresh and HPP treated samples, except for HPP3 application (Table 2). The limited impact of HPP treatment of the TAC could be explained by the stability of anthocyanins under high pressure conditions at moderate temperatures. Furthermore, the increasing tendency in the monomeric anthocyanins of HHP-treated fruit juice blends is related to the increased extractability of anthocyanins at extreme pressures as 600 MPa. HPP treatment can improve the cell permeability because of deprotonated charged groups as well as corrupted salt bridges and hydrophobic bonds in cell membranes, resulting in more accessible anthocyanins at higher pressure conditions (Cao et al., 2011). Although there was slight increase in antioxidant levels after PEF treatment, there was no significant difference evaluated in HPP, TT and fresh fruit juice blends (*p* *>* 0.05). Research studies demonstrated that PEF treatment could potentially improve the nutritional values of fruit juices with berries while enhancing the food safety. Approximately, 10 % anthocyanin content and 20 % phenolic content of blueberries increased after PEF treatment (Yu et al., 2017). Similar result was obtained in PEF-treated strawberry juice, which improved antioxidants and anthocyanin content (Odriozola-Serrano et al., 2009). This might be associated with electroporation process and disruption of cellular structures leading to extraction of phenolic compounds from berry fruits (Gabrić et al., 2018). On the other side, HPP showed positive effect on the stability of anthocyanin in juices (Waghmare, 2024).

In this study, bioactive compounds in the fruit juice blends were also analyzed by chromatographic methods to identify and quantify the individual phenolic acids, flavonoids, anthocyanins as well as vitamin C contents. Primarily, the individual phenolic acids and flavonoids in fruit juice blends were identified by UPLC-QTOF-MS/MS method according to their retention time, m/z value and MS/MS fragment ions (data not shown). The phenolic profile of the samples involves a hydroxybenzoic acid (benzoic acid), hydroxycinnamic acids (chlorogenic acid and cinnamic acid), a flavan-3-ol (catechin), a flavanone (hesperidin) and flavonols (rutin, quercetin, kaempferol, kaempferol-3-*O*-glucoside). The effect of processing techniques on the individual phenolic acids and flavonoids of fruit juice blends were indicated in Table 3. These findings indicated that HPP had positive impact on chlorogenic acid compared to PEF, TT and fresh fruit juice blends while PEF and TT samples had higher values than fresh ones (*p* *<* 0.05). Similarly, HPP and PEF increased chlorogenic acid in cranberrybush puree in the range of 6–7 % and 5.6–11 %, respectively (Ozkan, et al., 2022). In the current study, compared to TT, HPP at 600 MPa for 3 min enhanced the concentration of hesperidin and rutin from 449.1 ± 2.892 to 476.7 ± 4.752 mg/100 mL and 19.63 ± 1.153 to 30.45 ± 0.318 mg/100 mL, respectively (*p* *<* 0.05). Further study reported an increasing trend in individual flavonoid content, in terms of quercetin and kaempferol, in prickly pear juice after HPP under 400 to 550 MPa up to 16 min processing time at room temperature (Jiménez-Aguilar et al., 2015). Depending on the processing conditions, quercetin content of blended fruit juice either increased or remained constant after HPP and PEF treatments compared to TT samples. With TT, quercetin content significantly reduced from 13.32 ± 0.23 to 10.59 ± 0.42 mg/100 mL (*p* < 0.05). In a research study by (López-Giral et al., 2015) rutin concentration of the PEF treated different varieties of grape samples enhanced significantly. On the contrary, rutin content either reduced or not altered in fruit juice blends, In the present study, not only thermal but also non-thermal processing significantly decreased kaempferol-3-*O*-glucoside and kaempferol in fruit juice blends. It is noteworthy that higher kaempferol-3-*O*-glucoside and kaempferol was obtained after HPP at 500 MPa for 5 min and PEF at 100 kJ/L and 20 kV/cm treatment compared to thermal treated samples (*p* *<* 0.05).

**Table 3:** Changes in phenolic acid and flavonoid contents of fruit juice blends.^1^

Phenolic Compounds (mg/100 mL)	Control	TT	HPP1	HPP2	HPP3	PEF1	PEF2	PEF3
**Chlorogenic acid**	77.78 ± 1.57^d^	107.50 ± 1.79^c^	131.60 ± 1.12^ab^	136.32 ± 4.47^ab^	149.80 ± 6.91^a^	121.40 ± 1.70^bc^	110.16 ± 7.18^c^	114.61 ± 3.26^c^
**Hesperidin**	504.00 ± 2.85^a^	449.10 ± 2.89^c^	480.70 ± 4.05^b^	467.61 ± 5.38^c^	476.70 ± 4.75^b^	480.80 ± 0.86^b^	477.15 ± 1.52^b^	514.81 ± 10.47^a^
**Rutin**	22.11 ± 0.08^b^	19.63 ± 1.15^cd^	19.83 ± 0.59^c^	17.60 ± 0.42^d^	30.50 ± 0.32^a^	12.10 ± 0.07^f^	17.44 ± 0.31^d^	17.58 ± 0.41^d^
**Kaempferol-3-*O*-glucoside**	6.99 ± 0.15^a^	2.72 ± 0.16^f^	6.02 ± 0.01^b^	5.75 ± 0.53^bc^	4.47 ± 0.38^cd^	3.14 ± 0.10^ef^	3.30 ± 0.21^ef^	4.12 ± 0.09^de^
**Quercetin**	13.32 ± 0.23^ab^	10.59 ± 0.42^de^	13.92 ± 0.65^a^	11.77 ± 0.54^bcd^	9.76 ± 0.54^e^	10.73 ± 0.19^cde^	12.96 ± 0.68^abc^	9.16 ± 0.13^e^
**Kaempferol**	17.03 ± 0.14^a^	7.62 ± 0.27^d^	11.02 ± 0.72^b^	9.01 ± 0.01^cd^	8.80 ± 0.576^d^	5.88 ± 0.09^e^	10.41 ± 0.29^bc^	3.89 ± 0.28^f^

1 Data in the same row followed by the different letters are significantly (*p <* 0.05) different.

**Fig. 1:**
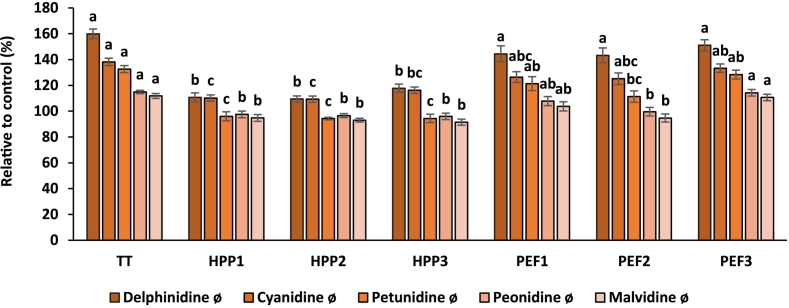
Effect of processing techniques on anthocyanin profile of fruit juice blends in percentage (%) relative to control sample (fresh). Different small letters on the bars represent statistically significant differences between treatments (*p <* 0.05).

Anthocyanin profile and relative contents grouped based on aglyca were compared with fresh sample (control) in percentage and presented in Fig. 1. Relative to fresh fruit juice blend (100 %), TT increased delphinidine, cyanidine, petunidine, peonidine, and malvidine levels up to 160 %, 138.1 %, 132.6 %, 114.9 % and 111.8 %, respectively. Furthermore, depending on the processing parameters, concentration of individual anthocyanins ranged from 143.2 % to 151.1 % for delphinidine, 125.1 % to 133.3 % for cyanidine and 111.3 % to 128.3 % for petunidine after PEF application to fruit juice blends. Even though HPP was not effective on anthocyanins, slight increase was observed in delphinidine and cyanidine up to 117.8 % and 116.3 % in 600 MPa for 3 min, 110.7 % and 110.2 % in 500 MPa for 5 min, 109.4 % and 109.2 % in 500 MPa for 10 min, respectively.

Most of the research studies proved that HPP preserved heat-sensitive molecule of ascorbate in beverages since this technique cannot break covalent bonds and rupture small molecules like vitamin C (Waghmare, 2024). In citrus fruits, TT caused most degradation of vitamin C through oxidative reaction compared to HPP (Barba et al., 2010; Cheng et al., 2020) Nevertheless, in the current study (Table 2), application of HPP under different conditions did not significantly affect the vitamin C content of fruit juice blend compared to fresh and TT alternatives (*p* *>* 0.05). In consistent to this study, researchers found no loss in vitamin C content in strawberry juice when they evaluated the efficacy of HPP and TT (Aaby et al., 2018). Similarly, no significant difference was observed in apple juice after HPP at 400 MPa for 3 min and TT at 75 °C for 15 s (Wibowo et al., 2019). Therefore, citrus fruits/juice alone might more sensitive to heat preservation techniques than other fruits/juice types. In this research study, PEF treatment significantly reduced vitamin C content of fruit juice blends from 23.75 ± 0.70 mg/100 mL to 19.43 ± 0.88 mg/100 mL (PEF1), 18.78 ± 0.33 mg/100 mL (PEF2), 18.38 ± 0.45 mg/100 mL (PEF3) when compared to TT samples (*p* *<* 0.05). However, there was no significant difference observed in fruit juice blend compared to fresh samples when processing parameters in terms of specific energy input and electric field strength reduced from 120 to 100 kJ/l and 24 to 20/15 kV/cm, respectively. These observations are consistent with PEF treated orange-carrot juice (Torregrosa et al., 2006), watermelon juice (Oms-Oliu et al., 2009), and strawberry juice (Odriozola-Serrano et al., 2009). From these data it can be inferred that active form of vitamin C can be maintained by PEF technology due to inactivation of some oxidative enzymes and prevention from oxidation (Faisal Manzoor et al., 2021).

### 3.3 Effect of processing on the bioaccesibility of bioactive compounds in fruit juice blends

According to the results obtained from section 3.2., the highest bioactive substances and antioxidant capacity values were obtained at HPP3 for high pressure and PEF3 for pulsed electric field applications. Therefore, only samples processed under these conditions as well as control group and TT-treated group of samples were used in the subsequent *in vitro* gastrointestinal digestion and shelf-life studies. The effects of non-thermal (HPP and PEF) and thermal (TT) processing applied under different treatment conditions on TPC, TFC, TAC and AOX of fruit juice blends were shown in Table 4. Findings demonstrated that bioactive contents and antioxidant capacities of the samples were generally decreased during digestion. Food processing had a diverse influence on the retention of phenolic substances from fruit juice blends, depending on the type of the processing and treatment intensity. A statistically remarkable bioaccessibility values were obtained for PEF3 sample in terms of TPC (37.73 %) and TFC (60.28 %) compared to HPP3, TT and control samples, whereas there was no significant difference observed in TPC and TFC values of HPP3-treated sample (*p <* 0.05) since both HPP and PEF increased the extraction rate thereby bioaccessibility rate. These outcomes were in line with the results from Rodríguez-Roque et al. (2015), which found demonstrated that HPP (400 MPa for 5 min) and PEF (35 kV/cm) treatments resulted in a significant improvement for the bioaccessibility of TPC and TFC in fruit juice samples. In another study, it was found that pomegranate juice had higher bioaccessible bioactive compounds (28.76 % for TPC, 32.59 % for CUPRAC, 35.26 % for DPPH) after thermosonication rather than control or thermal pasteurized samples (Yıkmış et al., 2022). On the other side, Barba et al. (2017) declared ascended or descended bioaccessibility of bioactive compounds after thermal treatment, depending on the food matrix and bioactive substance.

**Table 4:** Investigation of changes in the bioactive content and antioxidant capacity of fruit juice blends during *in vitro* gastrointestinal digestion.

Treatment^1^	UD	GD	ID	Bioaccessibility (%)
	**TPC (mg GAE/100 mL)**			
**Control**	92.36 ± 7.07^aA^	43.81 ± 5.76^bcB^	32.08 ± 1.65^bC^	34.92 ± 2.95^ab^
**TT**	95.13 ± 8.21^aA^	41.40 ± 5.12^cB^	30.05 ± 2.76^bC^	31.79 ± 2.87^b^
**HPP3**	94.42 ± 10.52^aA^	48.50 ± 5.40^abB^	32.92 ± 3.97^bC^	35.21 ± 3.68^ab^
**PEF3**	98.99 ± 10.78^aA^	51.09 ± 3.07^aB^	37.00 ± 3.86^aC^	37.73 ± 3.89^a^

	**TFC (mg RE/100 mL)**			
**Control**	24.59 ± 0.30^cA^	11.45 ± 0.95^cC^	12.76 ± 0.14^bB^	52.12 ± 0.36^b^
**TT**	26.96 ± 1.06^bA^	12.70 ± 1.95^abB^	14.09 ± 2.04^bB^	52.33 ± 2.05^b^
**HPP3**	26.90 ± 0.50^bA^	12.19 ± 0.28^bcC^	16.16 ± 1.20^aB^	60.09 ± 1.10^a^
**PEF3**	30.02 ± 0.91^aA^	13.60 ± 0.46^aC^	18.08 ± 1.50^aB^	60.28 ± 1.85^a^

	**TAC (mg C3G/L)**			
**Control**	58.22 ± 4.41^cA^	47.68 ± 6.10^bB^	28.06 ± 1.82^bC^	45.43 ± 0.68^b^
**TT**	70.16 ± 2.15^aA^	42.03 ± 4.28^bB^	21.67 ± 2.07^cC^	30.91 ± 0.96^c^
**HPP3**	65.92 ± 2.26^bA^	49.11 ± 4.02^bB^	35.87 ± 3.84^aC^	54.47 ± 1.83^a^
**PEF3**	74.20 ± 8.12^aA^	58.52 ± 10.17^aB^	37.37 ± 4.88^aC^	50.86 ± 5.44^ab^

	**CUPRAC (mg TE/100 mL)**			
**Control**	189.85 ± 32.11^aA^	94.21 ± 6.33^bcB^	88.39 ± 11.25^aB^	47.54 ± 7.00^ab^
**TT**	199.55 ± 30.20^aA^	88.82 ± 6.74^cB^	74.83 ± 6.43^bB^	38.19 ± 5.54^b^
**HPP3**	186.66 ± 16.61^aA^	101.67 ± 8.18^aB^	89.57 ± 6.76^aC^	48.30 ± 4.24^a^
**PEF3**	205.02 ± 29.23^aA^	99.55 ± 7.44^abB^	92.37 ± 8.99^aB^	45.81 ± 6.39^ab^

	**DPPH (mg TE/100 mL)**			
**Control**	99.74 ± 11.93^abA^	36.73 ± 5.68^aB^	39.48 ± 9.80^aB^	40.05 ± 4.71^ab^
**TT**	109.79 ± 6.92^aA^	38.94 ± 6.71^aB^	35.49 ± 6.97^aB^	32.43 ± 2.01^c^
**HPP3**	88.91 ± 10.52^bA^	41.08 ± 7.90^aB^	40.43 ± 6.25^aC^	45.99 ± 5.32^a^
**PEF3**	98.75 ± 7.05^abA^	42.83 ± 7.74^aB^	35.55 ± 4.01^aC^	36.15 ± 2.49^bc^

TPC: Total Phenolic Content, TFC: Total Flavonoid Content, TAC: Total Anthocyanin Content. GAE: Gallic acid equivalents, RE: Rutin equivalents, C3G: Cyanidin-3-*O*-glucoside equivalents, CUPRAC: Cupric ion reducing antioxidant capacity, DPPH: 2,2-diphenyl-1-picrylhydrazyl, n.d.: not detected. UD: pre-digestion; GD: gastric phase; ID: intestinal phase.

1 Data in the same column followed by the different small letters are significantly (*p <* 0.05) different. Data in the same row followed by the different capital letters are significantly (*p* < 0.05) different.

The changes in the individual phenolic content of the samples in the gastric and intestinal phases during *in vitro* gastrointestinal digestion are given in Table 5. Similar to those of spectrophotometric measurements, individual phenolic compounds also exhibited declining trend throughout digestion. According to the results obtained from the gastric phase, chlorogenic acid, rutin and kaempferol were found to be the highest in the PEF3-treated sample, while hesperidin was the highest in the TT-treated sample (*p* *<* 0.05). According to the results obtained from the intestinal phase, while chlorogenic acid, hesperidin, kaempferol-3-*O*-glucoside, quercetin and kaempferol were obtained as the highest in the HPP3-treated sample, TT-treated sample showed enhanced hesperidin and rutin (*p* *<* 0.05). Besides, quercetin was completely degraded in TT-treated sample during the digestive process and it could not be detected in the intestinal phase. Similarly, the bioaccessibility of quercetin was reported to be very scarce, since quercetin has low water solubility and crystalline state in water (Ozkan, Franco, et al., 2022).

**Table 5:** Changes in the individual phenolic content of fruit juice blends during *in vitro* gastrointestinal digestion (mg/100 mL).

**Treatment**^1^	**UD**	**GD**	**ID**	**Bioaccessibility (%)**
**Chlorogenic acid**				
**Control**	77.78 ± 1.57^cA^	56.82 ± 0.58^dB^	55.51 ± 0.36^dB^	71.04 ± 0.46^a^
**TT**	107.50 ± 1.79^bA^	77.69 ± 0.49^bB^	67.37 ± 0.26^cC^	62.50 ± 0.24^b^
**HPP3**	149.8 ± 6.91^aA^	72.80 ± 0.59^cB^	80.90 ± 0.64^aB^	53.70 ± 0.42^d^
**PEF3**	114.61 ± 3.26^bA^	86.62 ± 1.15^aB^	69.91 ± 0.64^bC^	60.60 ± 0.56^c^

**Hesperidin**				
**Control**	504.00 ± 2.85^aA^	351.10 ± 0.81^bB^	285.70 ± 4.02^bC^	56.62 ± 0.10^c^
**TT**	449.10 ± 2.89^cA^	362.60 ± 3.14^aB^	329.20 ± 6.49^aC^	73.28 ± 0.03^a^
**HPP3**	476.70 ± 4.75^bA^	312.20 ± 1.55^cB^	311.41 ± 0.98^aB^	65.28 ± 0.06^b^
**PEF3**	514.81 ± 10.47^aA^	346.20 ± 4.41^bB^	288.42 ± 2.38^bC^	55.98 ± 0.06^d^

**Rutin**				
**Control**	22.11 ± 0.08^bA^	7.15 ± 0.11^cC^	11.41 ± 0.07^bB^	50.68 ± 1.31^b^
**TT**	19.63 ± 1.15^cdA^	7.07 ± 0.05^cC^	11.93 ± 0.05^aB^	60.70 ± 0.11^a^
**HPP3**	30.50 ± 0.32^aA^	8.03 ± 0.19^bB^	9.08 ± 0.06^cB^	29.64 ± 0.19^d^
**PEF3**	17.58 ± 0.41^dA^	9.09 ± 0.07^aB^	7.32 ± 0.23^dC^	40.73 ± 1.29^c^

**Kaempferol-3-*O*-glucoside**				
**Control**	6.99 ± 0.15^aA^	2.24 ± 0.18^aB^	1.81 ± 0.13^bB^	25.75 ± 0.20^d^
**TT**	2.72 ± 0.16^cA^	2.51 ± 0.36^aA^	2.26 ± 0.10^abA^	81.99 ± 1.56^a^
**HPP3**	4.47 ± 0.38^bA^	3.12 ± 0.09^aB^	2.54 ± 0.18^aB^	56.38 ± 0.63^b^
**PEF3**	4.12 ± 0.09^bA^	2.52 ± 0.20^aB^	2.00 ± 0.14^abB^	46.84 ± 2.40^c^

**Quercetin**				
**Control**	13.32 ± 0.23^aA^	8.74 ± 0.52^abB^	2.55 ± 0.18^bC^	18.96 ± 0.27^b^
**TT**	10.59 ± 0.42^bA^	9.27 ± 0.19^aB^	n.d.	n.d.
**HPP3**	9.76 ± 0.54^bcA^	7.23 ± 0.19^bB^	6.21 ± 0.15^aB^	63.58 ± 0.07^a^
**PEF3**	9.16 ± 0.13^bcA^	7.82 ± 0.58^bB^	1.74 ± 0.04^cC^	18.78 ± 0.31^b^

**Kaempferol**				
**Control**	17.30 ± 0.14^aA^	9.67 ± 0.47^cB^	4.63 ± 0.26^aC^	26.68 ± 0.12^d^
**TT**	7.62 ± 0.27^bB^	16.13 ± 0.09^aA^	3.84 ± 0.11^bC^	50.13 ± 0.37^c^
**HPP3**	8.80 ± 0.57^bB^	11.49 ± 0.35^bA^	4.90 ± 0.07^aC^	55.17 ± 0.72^b^
**PEF3**	4.12 ± 0.09^cB^	15.79 ± 0.56^aA^	3.89 ± 0.28^bB^	93.33 ± 1.54^a^

1 Data in the same column followed by the different small letters are significantly (*p* *<* 0.05) different. Data in the same row followed by the different capital letters are significantly (*p* *<* 0.05) different. UD: pre-digestion; GD: gastric phase; ID: intestinal phase. n.d.: not detected.

Regarding the bioaccessibility of individual phenolic compounds, the bioaccessibility values were calculated to be in the range of 53.70–71.04 % for hydroxycinnamic acid (chlorogenic acid), 55.98–73.28 % for flavanone (hesperidin), and 29.64–60.70 %, 25.75–81.99 %, 18.78–63.58 % and 26.68–93.33 % for flavonols in terms of rutin, kaempferol-3-*O*-glucoside, quercetin, kaempferol, respectively, for control and thermal- or non-thermal treated fruit juice blends, depending on the type of the treatment. The results of the current study were comparable with the outputs of the research by Van de Velde et al. (2022), in which the bioaccessibility values were calculated to be in the range of 8–24 % for flavanones (naringenin-7-*O*-rutinoside and hesperitin-7-*O*-rutinoside) and 15–90 % for flavonols (kampferol-3-*O*-glucoside, quercetin-3-*O*-glucoside and kampferol-3-*O*- glucuronide) as well as 7 % for chlorogenic acid and 35 % for elleganic for unpasteurized and thermal pasteurized fruit smoothies. In the literature, it was reported that mild or high temperature levels may favor the recovery of phenolic compounds after digestion, due to alteration of food matrix related factors. These results were in line with those reported by Ozdemirli and Kamiloglu (2024) that the release of some of the flavonoids (primarily hesperidin and narirutin) throughout *in vitro* gastrointestinal was increased owing to thermal treatment to the orange peel, destroying the cell walls and promoting the liberation of flavonoids. In this context, the potent retention of some of the phenolics may also be related to their enhanced solubility (Ribas-Agustí et al., 2018), as indicated in this work for the hesperidin, rutin and kaempferol-3-*O*-glucoside when compared to those of non-thermal applications. Overall, inconsistency in the results in the bioaccessibility of individual phenolic compounds might be due to their chemical properties, including solubility, hydrophobicity, molecular weight and isomer configuration (Barba et al., 2017). Regarding the effect of food processing, the release, transformation and absorption of phenolic compounds may be modulated according to the type and intensity of the technology to be used (Verkempinck et al., 2020). On one hand, food processing could split the bonds between proteins and phenolic compounds, hence enhancing their content before and after digestion. On the other hand, heat treatment during food processing may reduce the level of bioactive compounds depending on the temperature and duration (Rodríguez-Roque et al., 2020).

### 3.4 Effect of processing on the shelf-life of fruit juice blends

**Fig. 2:**
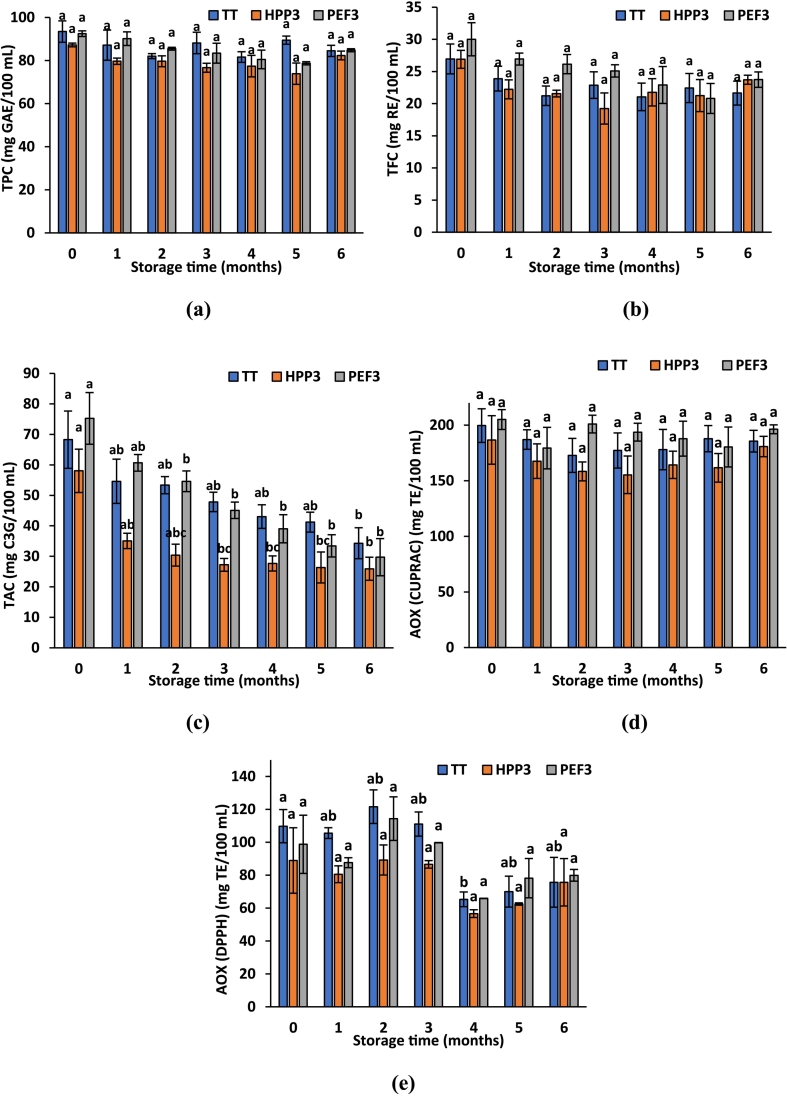
Effect of storage time on (a) total phenolic content, (b) total flavonoid content, (c) total anthocyanin content, (d) total antioxidant capacity by CUPRAC and (e) DPPH of TT-, HPP3- and PEF3-treated samples. Different small letters on the bars represent statistically significant differences during storage for each treatment (*p <* 0.05).

In order to examine the storage stability of HPP3, PEF3 and TT treated samples, the samples were packaged in PET bottles and stored at 4 °C for 6 months. The effects of food processing methods on TPC, TFC, TAC and AOX of fruit juice blend samples during the storage period are given in Fig. 2 (a-e). According to the results obtained, it was observed that the TPC content of the samples tended to decrease during the 6-month storage period (Fig. 2a). However, this decrease was not statistically significant. When the samples were compared among themselves, no significant difference was found between the samples except for the 1st month. When the changes in TFC content of the samples during storage was analyzed, in general, the lowest value was found in HPP-treated samples every month and the lowest value was reached in the 3rd month. It was also observed that the TFC content of the samples tended to decrease during the 6-month storage period (Fig. 2b). When the total monomeric anthocyanin contents of the blended fruit juices were evaluated during the storage period, similar results were obtained and the lowest value was found in the samples treated with HPP every month. In addition, there was a significant decrease in the total monomeric anthocyanin content (TAC) of each sample during storage (Fig. 2c). Similarly, significant reduction in TAC was observed in strawberry puree after HPP application under different pressures and heat treatment as well (Aaby et al., 2018). When the change in the antioxidant capacities of the blended juice samples during the storage period was examined, it was found that the antioxidant capacities of the samples, measured by CUPRAC (Fig. 2d) and DPPH (Fig. 2e) assays, tended to decrease during the 6-month storage period, as in TPC, TFC and total monomeric anthocyanin contents, and the lowest value was found in the samples treated with HPP every month. When analyzed in detail, the lowest CUPRAC antioxidant capacity value was observed in the 3rd month in HPP and PEF treated samples. These results were consistent with the finding that HPP and PEF resulted in similar antioxidant activities at the end of storage time as in strawberry juice samples (Yildiz et al., 2021). While evaluating the effects of food processing applications on the antioxidant capacity of the food products during storage, it should be taken into consideration that nonphenolic antioxidants such as vitamin C may also contirbute to the overall antioxidant activity of the samples. This reason for the variations in antioxidant capacity results could be attributed to the alterations in the results of vitamin C as well as individual anthocyanins.

**Fig. 3:**
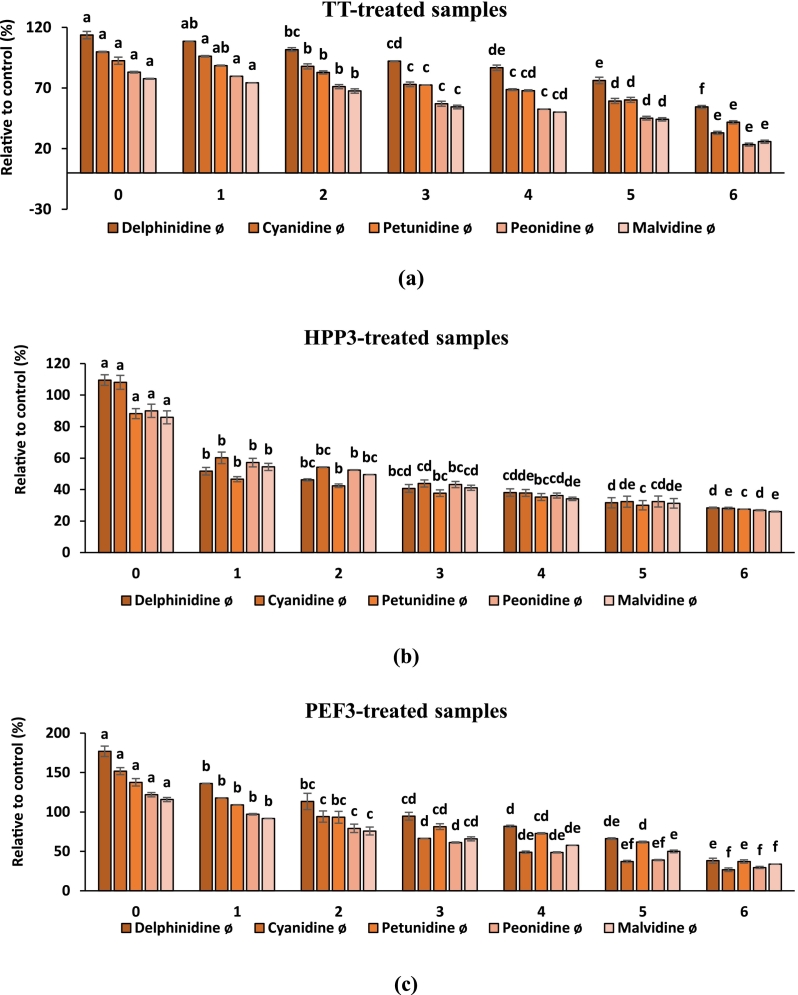
Changes in anthocyanin content of (a) TT, (b) HPP-3 and (c) PEF3-treated samples during storage according to their aglycones relative to control (%). Different small letters on the bars represent statistically significant differences over time within each individual anthocyanidin (*p <* 0.05).

During the shelf-life, changes in the anthocyanin profile and relative contents grouped based on aglycones were compared with fresh sample (control) in percentage and presented in Fig. 3. At the end of six months of storage, relative to fresh fruit juice blend (100 %), delphinidine significantly reduced from 113.63 to 54.36 %, 109.54 to 28.41 % and 176.94 to 38.13 % after TT, HPP3 and PEF3 treatments, respectively. Similar trend was determined in cyanidine, petunidine, peonidine, and malvidine concentrations of fruit juice blends after thermal and non-thermal treatments during the storage period. Besides, among others, delphinidin content was found to be the highest in TT treated sample when compared to other individual anthocyanin contents.

**Fig. 4:**
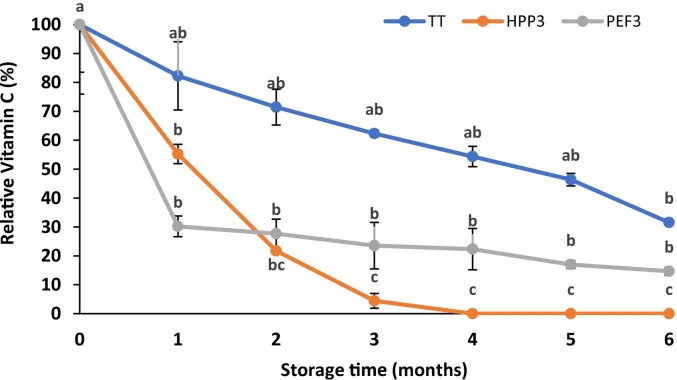
Changes in Vitamin C of TT-, HPP-3 and PEF3-treated samples during storage. Different small letters on the bars represent statistically significant differences over time for each treatment (*p <* 0.05).

Relative vitamin C content of TT, PEF3 and HPP3 treated fruit juice blends during the six months of refrigerated storage was shown in Fig. 4. After one month of storage, relative vitamin C content of samples was 82.25 %, 55.22 % and 30.21 % for TT, HPP3 and PEF3, respectively. Sharp decrease was observed in HPP3 treated samples after two months of storage and then reached to minimum level in three months. These results were consistent with the finding from the research study by (Yildiz et al., 2021). The vitamin C content of HPP treated strawberry puree was not detectable after 35 days of refrigerated storage, indicating longer storage time was not convenient for vitamin C content of the samples. Even though, there was reducing trend in TT samples during the six months of storage, TT provided higher level of vitamin C content compared to HPP3 and PEF3. Vitamin C content of PEF3 treated fruit juice blends remained constant from 2 to 6 months of storage (*p* *>* 0.05).

## 4 Conclusion

In this research, we aimed to unveil the influence of HPP and PEF applications as well as conventional thermal pasteurization on the content, bioaccessibility and stability of selected bioactive compounds in formulated fruit juice blends (21 % kiwi, 10.5 % mango, 37 % orange, 31.5 % blueberry). This study resulted in variable effects on the physicochemical properties as well as the level of phenolic acids, flavonoids, anthocyanins, vitamin C and antioxidant potential, depending on the type of treatment and operational conditions. In detail, HPP treatment at 600 MPa/3 min and PEF treatment at 120 kJ/L-24 kV/cm showed the greatest retention of bioactive contents. On the other side, PEF treatment at the same intensity encouraged the *in vitro* availability of TPC, TFC and TAC. Regarding the stability of bioactives during shelf-life, it could be concluded that while non-thermal and thermal-treated samples showed similar trends in terms of TPC, TFC, TAC and AOX, HPP3 processing led to lower individual anthocyanin and vitamin C content. Considering the entire outcomes of the present study, HPP and PEF treatments can be suggested as potential non-thermal processing methods for fruit juice blends. As future prospects, it would be helpful to understand the overall bioactivity of the phenolic acids, flavonoids, anthocyanins and vitamin C as well as their metabolites by applying the *in vitro* or *in vivo* bioavailability studies.

## CRediT authorship contribution statement

**Gulay Ozkan:** Writing – review & editing, Writing – original draft, Methodology, Data curation, Conceptualization. **Manolya Eser Oner:** Writing – review & editing, Writing – original draft, Methodology, Data curation, Conceptualization. **Annik Fischer:** Writing – review & editing, Methodology, Data curation. **Andreas Juadjur:** Writing – review & editing, Methodology, Data curation. **Kemal Aganovic:** Writing – review & editing, Supervision, Funding acquisition. **Gerald Dräger:** Methodology, Data curation. **Esra Capanoglu:** Writing – review & editing, Supervision, Project administration, Conceptualization. **Tuba Esatbeyoglu:** Writing – review & editing, Visualization, Supervision, Project administration, Methodology, Funding acquisition, Conceptualization.

## Funding declaration

This research was supported by 2531 Bilateral Cooperation Programme with German Academic Exchange Service (DAAD - project number 57589815) and The Scientific and Technological Research Council of Türkiye (project number 121N393). The publication of this article was funded by the Open Access Fund of Leibniz Universität Hannover.

## Declaration of competing interest

The authors declare the following financial interests/personal relationships which may be considered as potential competing interests: Given her role as Editor, Tuba Esatbeyoglu, had no involvement in the peer-review of this article and has no access to information regarding its peer-review.

## Data Availability

Data will be made available on request.
